# Kinase-Inactivated EGFR Is Required for the Survival of Wild-Type EGFR-Expressing Cancer Cells Treated with Tyrosine Kinase Inhibitors

**DOI:** 10.3390/ijms20102515

**Published:** 2019-05-22

**Authors:** Rintu Thomas, Shivangi Srivastava, Rajasekhara Reddy Katreddy, Jason Sobieski, Zhang Weihua

**Affiliations:** Department of Biology and Biochemistry, College of Natural Science and Mathematics, University of Houston, Houston, TX 77204-5036, USA; rtthomas3@uh.edu (R.T.); shivangi91srivastava@gmail.com (S.S.); rajasekharareddy.nitw@gmail.com (R.R.K.); jason.sobieski@hotmail.com (J.S.)

**Keywords:** EGFR, tyrosine kinase inhibitor, dimerization, palmitoylation, kinase-independent function, resistance

## Abstract

Inhibiting the tyrosine kinase activity of epidermal growth factor receptor (EGFR) using small molecule tyrosine kinase inhibitors (TKIs) is often ineffective in treating cancers harboring wild-type EGFR (wt-EGFR). TKIs are known to cause dimerization of EGFR without altering its expression level. Given the fact that EGFR possesses kinase-independent pro-survival function, the role of TKI-inactivated EGFR in cancer cell survival needs to be addressed. In this study, using wt-EGFR-expressing cancer cells A549 (lung), DU145 (prostate), PC3 (prostate), and MDA-MB-231 (breast), we characterized the TKI-induced dimerization status of EGFR and determined the dependency of cells on kinase-inactivated EGFR for survival. We report that TKI-induced EGFR dimerization is dependent on palmitoylation and independent of its kinase activity, and that mutations of the cysteine residues known to be critical for EGFR’s palmitoylation abolished TKI-induced EGFR dimerization. Furthermore, TKI-induced EGFR dimerization is persistent in TKI-resistant cells, and inhibition of palmitoylation by 2-bromopalmitate, or targeted reduction of the kinase-inactivated EGFR by siRNA or by an EGFR-downregulating peptide, are lethal to TKI-resistant cancer cells. This study suggests that kinase-inactivated EGFR remains to be a viable therapeutic target for wt-EGFR cancers and that inhibiting palmitoylation or downregulating EGFR may overcome TKI resistance.

## 1. Introduction

The epidermal growth factor receptor (EGFR/ErbB1) is a receptor tyrosine kinase (RTK) of the ErbB family of proteins (ErbB1-4). EGFR undergoes homo or hetero asymmetric dimerization in response to ligand stimulation, which consequentially leads to EGFR autophosphorylation at key tyrosine residues in its intracellular domain that activates downstream signaling cascades regulating cell growth [1,2]. Alterations of EGFR (overexpression or kinase-activating somatic mutations) are common in cancers [3]. Overexpression of EGFR is associated with an increase in cancer cell survival, metastasis, invasion, resistance to chemotherapy, and poor prognosis [4,5]. Both monoclonal antibodies and small tyrosine kinase inhibitors (TKIs) to block/inhibit EGFR’s kinase activity have been developed as targeted therapies for EGFR-dependent cancers [1,2,5].

TKIs are quinazoline-derived small molecules that inhibit EGFR’s kinase activity by binding reversibly or irreversibly to the adenosine triphosphate (ATP) binding pocket in the kinase domain [6]. However, in clinic TKIs are only transiently effective in 10% to 40% of non-small cell lung cancer patients (NSCLC) with EGFR mutations [7,8,9,10]. Emergence of acquired resistances result in disease progression in all TKI-treated patients with a mean progression-free survival of 9 to 13 months [11]. Acquired resistances are often facilitated by molecular adaptations which can occur at two levels. The first and most common resistance mechanism is the development of somatic mutations of EGFR, such as T790M and C797S, that decrease sensitivity to TKI treatment [12]. The second type of resistant mechanism involves molecular alterations that lead to activation of alternative oncogenic pathways that regulate cellular growth and survival. These may include amplification of oncogenic RTK-c-Met, and gain-of-function mutations of Ras/Raf/MEK/extracellular signal regulated kinase (ERK), and the phosphatidylinositol-3-kinase (PI3K)/Akt signaling pathways [13,14,15,16,17,18,19]. In addition, cancers expressing wild-type EGFR (wt-EGFR) are often insensitive to TKIs [20,21,22,23], although TKIs are potent in inhibiting the kinase activity of wt-EGFR as well [24,25,26].

Studies have shown that EGFR possesses pro-survival function, independent of its canonical kinase activity, as a scaffold protein interacting and stabilizing key survival proteins such as sodium/glucose co-transporter (SGLT1) to maintain glucose uptake of cancer cells [4], p53-upregulated modulator of apoptosis (PUMA) to repress apoptosis [27], lysosomal-associated transmembrane protein 4B (LAPTM4B) to promote pro-survival autophagy [28], fatty acid synthase (FASN) to promote de novo fatty acid synthesis [29], system x_c_−antiporter to maintain cysteine import [30], and mammalian target of rapamycin complex 2 (mTORC2) to inhibit mitophagy [31]. In addition, recent study has found that the C-terminus non-phosphorylatable EGFR mutant is oncogenic [32], clearly suggesting that the oncogenic potential of EGFR resides in its kinase-independent functions. Moreover, downregulation of EGFR protein causes cancer cell death through induction of mitophagy [31]. In this regard, we speculate that kinase-inactivated EGFR in TKI-treated cancer cells may be required for cell survival [33] and facilitate resistance. Therefore, targeting the kinase-inactivated EGFR in TKI-treated/resistant cancer cells holds therapeutic value.

Gefitinib and erlotinib, two of the most commonly used EGFR-TKIs, are known to cause EGFR dimerization by binding to the active conformation of EGFR while effectively blocking its kinase activity. This type of ligand-independent TKI-induced dimerization, also known as quasi-dimerization, is mediated by the kinase interface and does not depend on extracellular domain of EGFR [34,35,36]. Thus, following TKI treatment, EGFR remains in cells in a kinase-inactive state (i.e., the function of EGFR is completely shifted to its kinase-independent phase), however, its impact on cell survival is unknown. There are two possibilities for targeting the kinase-inactivated EGFR in TKI-treated cells; target the process of TKI-induced EGFR dimerization, or downregulate the kinase-inactivated EGFR proteins. Therefore, identifying the biochemical nature of TKI-induced EGFR dimerization is critical for evaluating its value as a therapeutic target. In addition, knowing the impact of complete removal of kinase-inactivated EGFR on survival of EGFR-TKI resistant cells will help to elucidate its role in facilitating resistance.

In this study, using four types of wt-EGFR-positive cancer cell lines—A549 (lung), DU145 (prostate), PC3 (prostate), and MDA-MB-231 (breast)—we investigated the biochemical nature of the TKI-induced EGFR dimerization, and tested the effects of either chemically disrupting the TKI-induced EGFR dimerization or downregulating total EGFR protein on TKI-resistant cancer cells. We found that TKI (gefitinib and erlotinib)-induced EGFR dimerization is dependent on palmitoylation and independent of kinase activity, mutations at cysteine residues known to be critical for EGFR’s palmitoylation abolished TKI-induced EGFR dimerization, TKI-induced EGFR dimerization is persistent in TKI-resistant cells, and inhibition of palmitoylation or targeted reduction of EGFR are lethal to TKI-resistant cancer cells.

## 2. Results

### 2.1. Epidermal Growth Factor Receptor (EGFR) Dimerization in Response to Acute Treatment of Tyrosine Kinase Inhibitors (TKIs)

**Figure 1:**
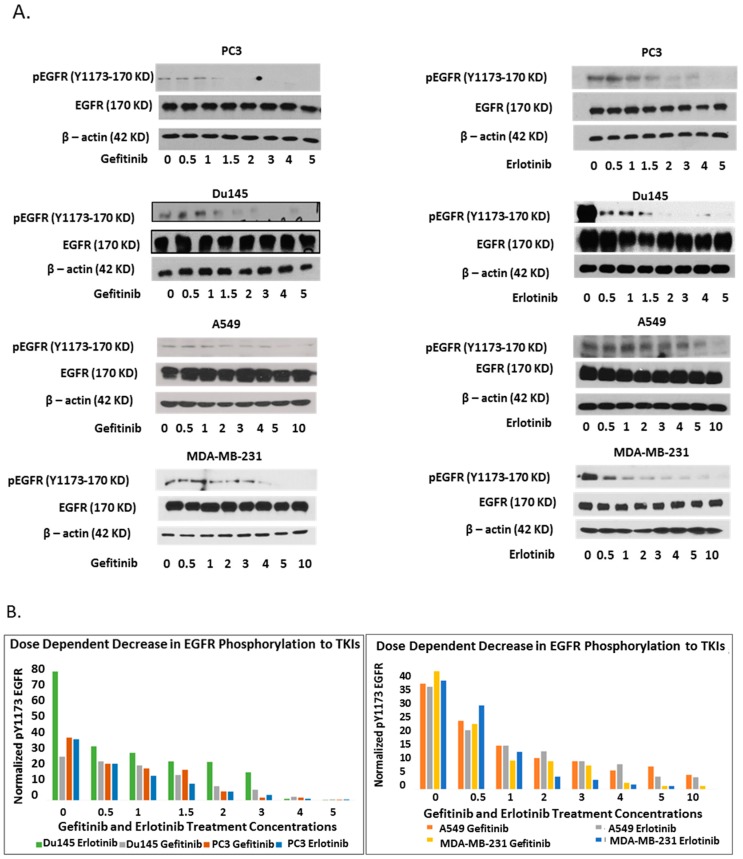

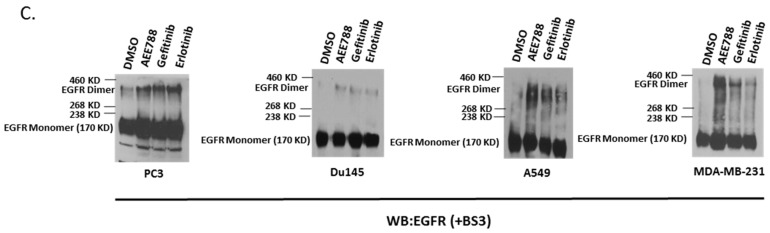
Acute treatment with tyrosine kinase inhibitors (TKIs) induce membrane-tethered epidermal growth factor receptor (EGFR) dimerization. (**A**) Gefitinib and erlotinib inhibit EGFR’s kinase activity in a dose-dependent manner. EGFR-positive cells (PC3, Du145, A549, and MDA-MB-231) were treated with an increasing concentration of either gefitinib or erlotinib acutely for 24 h. The cell lysates were collected for Western blot; (**B**) graphs represent the quantification of pY1173 EGFR from panel (**A**) and are normalized to the corresponding EGFR to β-actin fold change for all the treatments; (**C**) TKIs induce EGFR dimerization. PC3 and Du145 were treated with 5 μM concentration of TKIs (AEE788, gefitinib, and erlotinib), and A549 and MDA-MB-231 were treated with 10 μM of TKI in addition to vehicular control in serum-free media (absence of ligands) for 24 h. The degrees of EGFR dimerization due to TKI treatments were analyzed using membrane crosslinking reagent BS3. The cell lysates were resolved on sodium dodecyl sulfate polyacrylamide gel (SDS-PAGE) in reducing conditions followed by Western blot.

The ability of first generation TKIs, gefitinib and erlotinib, to effectively inhibit EGFR’s kinase activity were assessed in different wt-EGFR-positive cancer cells: prostate cancer cells PC3 and Du145, lung cancer cell A549, and breast cancer cell MDA-MB-231. The cells were treated with increasing doses of TKI (0.5 to 10 µM) for 24 h and EGFR’s phosphorylation status was assessed as a marker of its kinase activity using Western blot analysis. Both gefitinib and erlotinib were able to markedly decrease EGFR’s phosphorylation in a dose-dependent manner without significantly affecting EGFR protein levels in all cell lines (Figure 1A). However, the maximum kinase abrogating dosage of gefitinib and erlotinib varied between cell types ranging from 5 µM for PC3 and Du145 to 10 µM for A549 and MDA-MB-231 cells. All the EGFR-positive cell lines tested showed dose-dependent sensitivity to TKI treatment (Figure 1B).

It has been found that first generation TKIs can induce EGFR dimerization [34,35]. We then determined that TKIs at their kinase-abrogating dose could also cause EGFR dimerization. All the cell lines were incubated with kinase-abrogating doses of gefitinib, erlotinib, and AEE788 (also a reversible TKI) [37] acutely for 24 h in serum-free media followed by live cell chemical cross-linking using BS3 (bis[sulfosuccinimidyl]suberate), and the degree of dimerization was analyzed by immunoblotting. BS3 is a membrane-impermeable amine-to-amine homo-bifunctional cross-linker that is able to capture EGFR’s dimerization status on the cell membrane [38]. We observed that all the TKIs caused EGFR dimerization while EGFR’s kinase activity was blocked (Figure 1C).

### 2.2. Kinase-Inactivated EGFR Dimers Increased in TKI Resistant Cells

**Figure 2:**
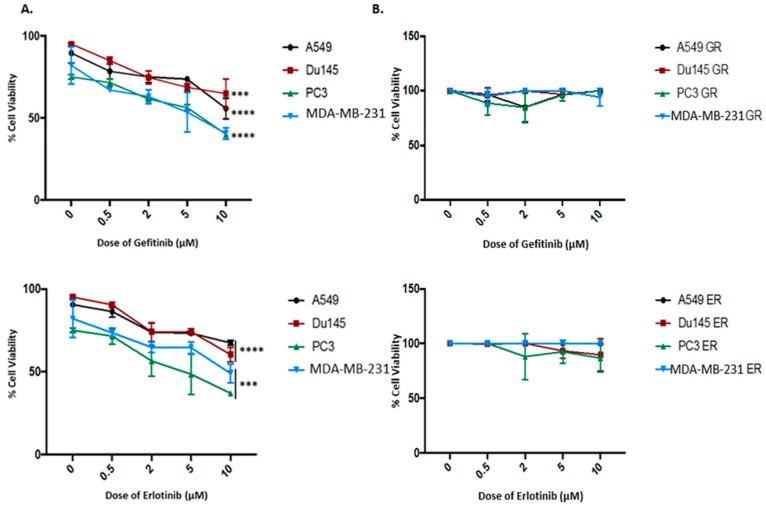

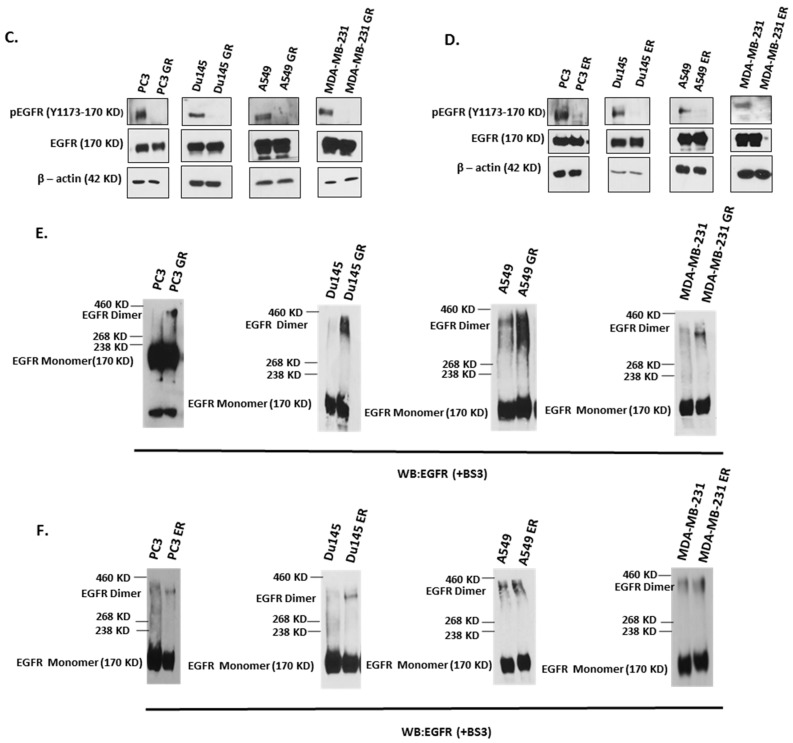
Increase in membrane-tethered EGFR quasi-dimers in TKI-resistant cells. (**A**) Evaluation of efficacy of wild-type EGFR (wt-EGFR)-positive cells to TKI treatments. EGFR-positive cell types were seeded on 6-well plates. Once the cells were 80% confluent, the cells were treated with increasing doses (lowest dosage was 0.5 μM and highest dosage was 10 μM) of gefitinib and erlotinib for 72 h in serum-free media. Equal volumes of cell suspension were incubated with 0.4% trypan blue to obtain 1:2 dilutions. Cells were counted using hemocytometer and cell viability was calculated. Results were triplicated ± SD and were pooled into treatment groups. One-way analysis of variance (ANOVA) and Tukey’s multiple comparison test were used for statistical analyses, *** *p* ˂ 0.001, **** *p* ˂ 0.0001; (**B**) Survival of gefitinib-resistant (GR) and erlotinib-resistant (ER) cells not affected by TKI treatments. All the GR cells (PC3 GR, PC3 ER, Du145 GR, Du145 ER, A549 GR) were treated with increasing dosage of gefitinib and the ER cells (A549 ER, MDA-MB-231 GR, MDA-MB-231 ER) were treated with increasing dosages of erlotinib for 72 h and cell proliferation was measured using MTT (Promega). Percent viable cells were calculated for each dosage against the vehicle (0.5% DMSO). Data are mean ± SEM with *n* = 3; (**C**,**D**) comparison of EGFR’s kinase activity (pEGFR) in chronically-treated GR and ER cells versus the non-treated parental cells; (**E**,**F**) TKI-induced membrane-tethered EGFR dimers persist in GR and ER cells. The degree of dimerization were analyzed in both GR and ER cells compared to the parental cells using membrane crosslinking agent BS3. The cell lysates were resolved on SDS-PAGE gel in reducing conditions followed by western blot.

We tested the impact of increasing dosage of TKIs on cell viability of non-treated (naïve) wt-EGFR-positive cancer cells. All the cancer cells were treated with increasing doses of either gefitinib or erlotinib (0.5 to 10 µM) for 72 h in serum-free media. Percent cell viability was evaluated using trypan blue exclusion assay. We observed there was a dose-dependent decrease in cell viability for all the EGFR-positive cells tested, however only the 10 µM dose (a non-physiologically relevant dose) reached statistical significance (Figure 2A). These results suggest that these wt-EGFR cells are relatively insensitive to TKIs.

To determine whether TKI-induced EGFR dimerization is involved in TKI resistance, we developed EGFR-TKI-resistant cells by exposing cells chronically to gefitinib or erlotinib for up to three months at the maximum tolerable concentration. Using MTT (3-(4,5-dimethylthiazol-2-yl)-2,5-diphenyltetrazolium bromide) cell proliferation assay, we evaluated the cell growth of both gefitinib-resistant (GR) and erlotinib-resistant (ER) cells treated with an increasing dosage (0.5 to 10 µM) of either gefitinib or erlotinib to assess their resistance to TKIs. The results revealed that the cell growth of both GR and ER cells was largely unaffected by treatments of TKIs at increasing doses (Figure 2B), which indicates that the GR and ER cells have acquired resistance to TKIs.

To determine the activity status of EGFR in the TKI-resistant cells, we measured the levels of phosphorylated EGFR (pEGFR) in these cells in comparison to the respective non-treated naïve cells. As shown in Figure 2C,D, there was no detectable pEGFR in the resistant cells, suggesting that the kinase activity of EGFR in the resistant cells was completely inactivated. We then compared the EGFR dimerization status of the TKI-resistant cells versus the non-treated parental cells. We observed that there was a significant increase in the levels of dimerized EGFR in the resistant cells (Figure 2E,F). These results indicate that EGFR continues to exist in its kinase-inactivated and dimerized status in chronically-induced TKI-resistant cells.

### 2.3. Inhibition of Palmitoylation Abolishes TKI-Induced EGFR Dimer Formation

**Figure 3:**
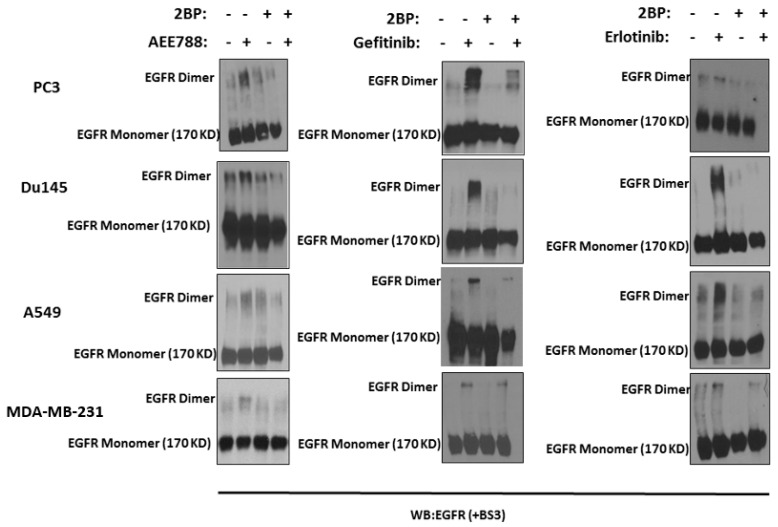
Inhibition of palmitoylation blocks TKI-induced EGFR dimerization. Cells were pretreated with 2-bromopalmitate (2-BP) at a concentration of 4 μM for 6 h in serum-free media. Following pretreatment, fresh media was added and the cells were treated with respective TKIs (AEE788. gefitinib, and erlotinib) at a final concentration of 5 μM for 24 h. The degree of EGFR dimerization were analyzed following membrane crosslinking using BS3. The cell lysates were resolved on SDS-PAGE gel in reducing conditions followed by Western blot.

Palmitoylation is an evolutionally-conserved global process which involves reversible lipid modification of proteins with a 16-carbon palmitate group, most commonly at cysteine residues and less frequently at serine (S) residues [39,40]. It has been previously reported that palmitoylation is critical for EGFR membrane localization, dimerization, and subsequent activation of EGFR [41,42]. To determine if palmitoylation is involved in TKI-induced EGFR dimerization, we first used 2-bromopalmitate (2-BP), an irreversible inhibitor of palmitoyl acyl transferases [43], in combination with TKIs to treat cells. As shown in Figure 3, TKI-induced EGFR dimerization was markedly reduced in cells pretreated with 2-BP. Fatty acid synthase (FASN) is a critical enzyme involved in de novo production of palmitate and involved in protein palmitoylation [41,44]. TKI-induced EGFR dimerization was also disrupted by a FASN inhibitor, cerulenin (Figure S1A). These results suggest that palmitoylation plays a crucial role in TKI-induced EGFR dimerization.

### 2.4. Mutations of Cysteine Residues Critical for EGFR Palmitoylation Abolished TKI-Induced Dimerization, and the Kinase Activity of EGFR Is Not Required for TKI-Induced Dimerization of EGFR

**Figure 4:**
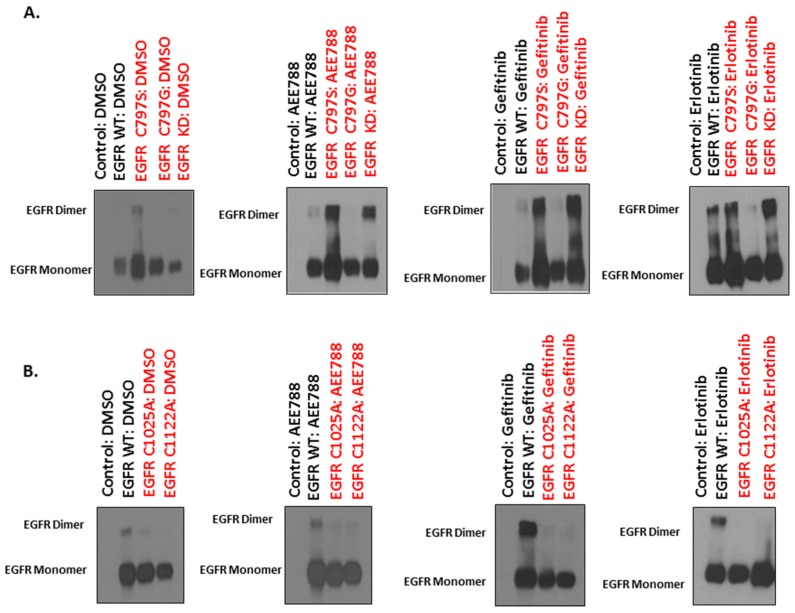
Mutations of palmitoylated cysteine in the kinase domain and C-terminal tail of EGFR blocks TKI-induced receptor dimerization. Upper panel: (**A**) HEK293 cells were transfected with negative control (no plasmid), and 100 ng of wt-EGFR, EGFR cysteine mutants (C797S and C797G) and EGFR kinase-dead (EGFR KD) mutant using polyethylene immine (PEI), respectively. Following 24 h post-transfection, the cells were treated with either DMSO or TKIs (AEE788, gefitinib, and erlotinib) at a concentration of 5 μM for 24 h in serum-free conditions. Cell lysates were collected in non-reducing conditions and resolved on SDS-PAGE gel followed by Western blot; Lower panel: (**B**) HEK293 cells were transfected with negative control (no plasmid), and 100 ng each of wt-EGFR, C1025A mutant and C1122A mutant EGFR using PEI. Following 24 h post-transfection, the cells were treated with either DMSO or TKIs (AEE788, gefitinib, and erlotinib) at a concentration of 5 μM for 24 h in serum-free conditions. Cell lysates were collected in non-reducing conditions and resolved on SDS-PAGE gel followed by Western blot.

Protein s-palmitoylation is the most common acylation observed in eukaryotic cells where key cysteine residues are covalently attached to a palmitoyl group via a sulfhydryl bond [39,40]. Several key cysteine residues involved in EGFR palmitoylation were identified in the intracellular domain of EGFR. Of these cysteine residues, the C797 (located in the kinase domain) has been identified to be conserved in kinase-active ErbB members (HER2 and HER4) and has also been proved critical for ligand-induced EGFR dimerization and subsequent activation [41]. To study the role of the C797 site in TKI-induced EGFR dimerization, we expressed the C797G mutant EGFR (cysteine (C) was mutated to glycine (G)) in HEK293 cells followed by TKI treatments in serum-free conditions. The degree of dimerization was assessed using immunoblotting in non-reducing conditions. It was found that the C797G mutant prevented TKI-induced dimerization of EGFR (Figure 4A). Further, two more palmitoylated cysteine sites, C1025 and C1122, were identified in the C-terminal tail of EGFR and mutated to alanine (A) [42]. We expressed the respective cysteine mutant versions of EGFR, C1025A and C1122A, in HEK293 to study the impact of these palmitoylated residues on TKI-induced dimerization. Following TKI treatments, we observed that both C1025A and C1122A also significantly impeded TKI-induced EGFR dimerization (Figure 4B). These results suggest all the three cysteine residues (C797, C1025, and C1122) are required for TKI-induced EGFR dimerization.

The C797S mutation in EGFR is a clinically acquired mutation responsible for resistance to the third generation TKIs that target the EGFR T790M mutant non-small lung cancers [45]. Palmitoylation at serine residues (also referred as o-palmitoylation) is less frequent compared to cysteine residues [39]. We therefore evaluated the effect of C797S mutation on TKI-induced EGFR dimerization. We expressed the EGFR C797S mutant in HEK293 cells followed by TKI treatments. It was found that the C797S mutation did not affect TKI-induced dimerization of EGFR (Figure 4A).

To evaluate if EGFR’s kinase activity is required for TKI-induced dimerization, we expressed a kinase-impaired (kinase dead, KD) EGFR mutant (R817M, arginine (R) was mutated to methionine (M)) [46] in HEK293 cells followed by TKI treatments. We found that TKIs induced dimerization of the KD-EGFR (Figure 4A). These results suggest that TKI-induced EGFR dimerization is independent of its kinase activity.

### 2.5. Both Inhibition of Palmitoylation and Targeted Reduction of EGFR Are Lethal to TKI-Resistant Cells

**Figure 5:**
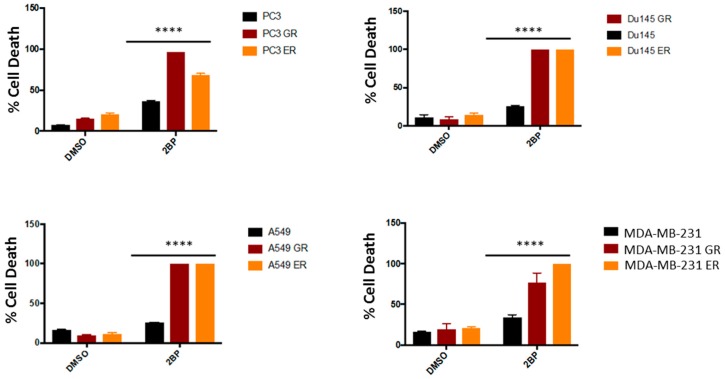
Inhibition of palmitoylation increases cytotoxicity in EGFR-TKI-resistant cells. GR and ER cells were seeded on 6-well plates along with respective non-treated parental controls. Once the cells were 80% confluent, the cells were treated with 2-BP (4 μm) for 72 h in serum-free media. Equal volumes of cell suspension was incubated with 0.4% trypan blue to obtain 1:2 dilution. Cells were counted using hematocytometer and percent cell death calculated. Results were triplicated ± SD and were pooled into treatment groups. One-way ANOVA and Tukey’s multiple comparison test were used for statistical analyses, **** *p* ˂ 0.0001.

The kinase-inactivated EGFR remains in TKI-resistant cells in two forms: monomer and palmitoylation-dependent dimer. However, the impact of these two forms of EGFR on TKI-resistant cells is unknown. The palmitoylation dependency of TKI-induced EGFR dimerization offers a targetable mechanism for overcoming TKI resistance. We used the palmitoylation inhibitor 2-BP to disrupt EGFR dimerization status to determine its impact on TKI-resistant cells. We assayed for the cytotoxicity induced by palmitoylation inhibitor 2-BP using the trypan blue exclusion assay. We observed that, irrespective of the cell lines tested, inhibition of palmitoylation using 2-BP resulted in higher cytotoxicity in both GR and ER cells compared to the parental cells (Figure 5). Similarly, impeding de novo palmitate synthesis by inhibiting FASN using cerulenin also gave the same result (Figure S2). These results suggest that palmitoylation is an important mediator that facilitates resistance to TKIs and is an effective therapeutic target for overcoming TKI resistance.

**Figure 6:**
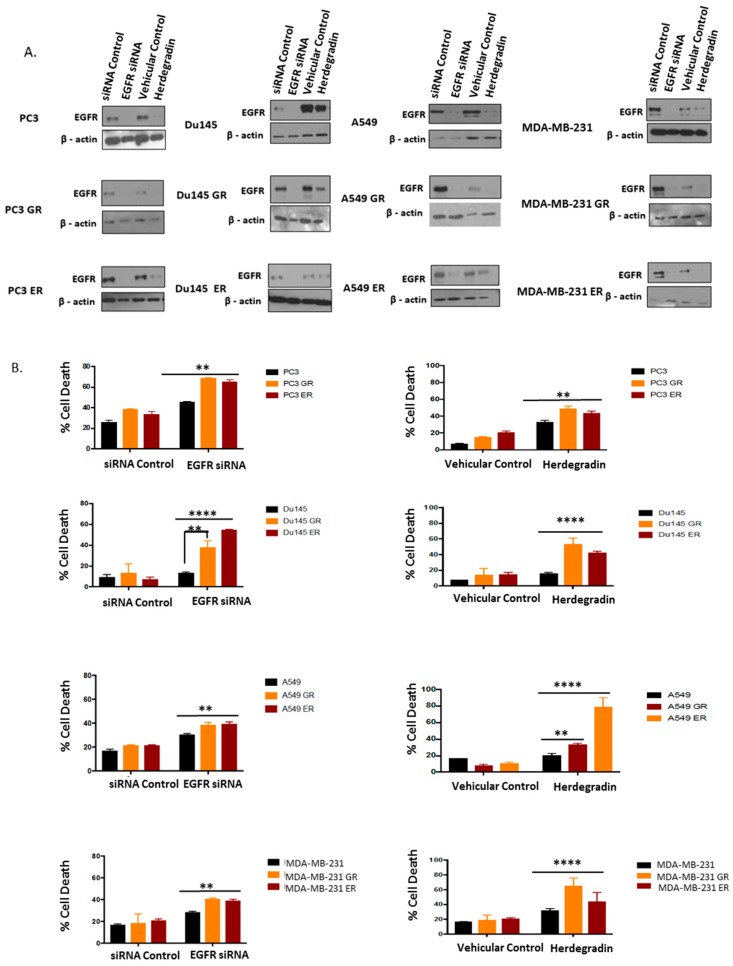
Targeted reduction of EGFR increases cytotoxicity in EGFR-TKI-resistant cells. (**A**) GR and ER cells were seeded on 12-well plates along with respective non-treated parental controls. Once the cells were 80% confluent, the cells were grouped into three experimental treatment groups: EGFR knockdown group transfected with EGFR specific siRNA compared against EGFR siRNA control, and cells treated with EGFR downregulating peptide-herdegradin compared against vehicular control. All the cells were treated for 72 h in serum-free media. The maximum tolerable concentrations of herdegradin were used (PC3, PC3ER, PC3GR: 2.5 μm, Du145, Du145 GR, Du145 ER: 20 μM, A549, A549ER, A549GR: 20 μM, MDA-MB-231, MDA-MB-231 GR, MDA-MB-231 ER: 20 μM) for treatment above which complete cell death was induced in both non-treated parental controls and resistant cells. Cell lysates following treatment were collected and Western blot analysis was conducted to examine EGFR expression; (**B**) GR and ER cells were seeded on 6-well plates along with non-treated parental controls. Once the cells were 80% confluent, the cells were assigned to three experimental groups: EGFR knockdown group transfected with EGFR specific SiRNA compared against EGFR siRNA control, and cells treated with EGFR downregulating peptide-herdegradin compared against the vehicular control. All the cells were treated for 72 h in serum-free media, following which percent cell death was calculated for each treatment group. The concentration of peptide herdegradin varied according to cell types (PC3, PC3ER, PC3GR: 2.5 μm, Du145, Du145 GR, Du145 ER: 20 μM, A549, A549ER, A549GR: 20 μM, MDA-MB-231, MDA-MB-231 GR, MDA-MB-231 ER: 20 μM). Equal volumes of cell suspension were incubated with 0.4% trypan blue to obtain 1:2 dilution. Cells were counted using hematocytometer and percent cell death calculated. Results were triplicated ± SD and were pooled into treatment groups. One-way ANOVA and Tukey’s multiple comparison test was used for statistical analyses, ** *p* ˂ 0.001, **** *p* ˂ 0.0001.

To further determine the role of kinase-inactivated EGFR in maintaining the survival of EGFR-TKI-resistant cells, we used both siRNA and peptide herdegradin [31] to knockdown/downregulate the kinase-inactivated EGFR in the TKI-resistant cells. As shown in Figure 6A,B, both siRNA and the peptide were able to downregulate EGFR expression and these treatments were cytotoxic to both GR and ER cells. These results indicate that kinase-inactivated EGFR in ER and GR cells is required for cell survival and remains a viable therapeutic target.

## 3. Discussion

The clinical benefits of EGFR TKI therapies are challenged by resistance. Several mechanisms have been identified associated with EGFR TKI resistance, which include occurrence of EGFR secondary mutations that hamper the inhibitory effect of TKI, and the activation of alternative pro-survival signaling pathways [47]. Besides EGFR’s kinase function, which is prominently implicated in cellular growth and proliferation [48], emerging evidence shows that EGFR possess kinase-independent function that is critical for survival of cancer cells [4,6,33]. In our study we observed that chronic treatment with TKIs caused EGFR to remain in a kinase-inactive and quasi-dimerized state. Therefore, the main purposes of this study were to assess the role of TKI-inactivated EGFR in the survival of TKI-resistant cells and to test whether the kinase-inactivated EGFR remains to be a viable target for therapy.

Conventionally, the anti-tumor effects induced by kinase inhibitors are associated with their ability to inhibit the kinase activity of EGFR [33]. In this study, we observed a substantial inhibition of EGFR kinase activity in chronically treated GR and ER cells compared to the control. With respect to EGFR receptor dynamics, we observed a substantial increase in membrane-tethered EGFR dimerization. However, the lack of response of GR and ER cells to increasing dosages of both gefitinib and erlotinib prompted us to speculate that the kinase-inactivated TKI-induced EGFR dimers have a non-catalytic scaffolding function that is critical for survival of TKI-resistant cells. In order to test this, we wanted to first identify the biochemical nature of the TKI-induced membrane-tethered EGFR dimers and the impact that ensues due to its disruption of cell survival.

Previous mutational studies have indicated that first generation TKIs such as erlotinib and gefitinib, which bind to active conformational state of EGFR, induce receptor dimerization by initiating kinase domain interface-mediated asymmetric interaction between two receptor monomers [49,50]. We demonstrate for the first time that inhibition of palmitoylation, a lipid post-translational modification, blocked TKI-induced EGFR dimerization, which suggests that the TKI-induced asymmetric interaction of EGFR’s tyrosine kinase domain is stabilized by palmitoylation. To further support our finding about the critical nature of palmitoylation on TKI induced EGFR receptor dimerization, we mutated identified palmitoylated cysteine residues (C797G, C1025A, and C1122A) located in the kinase domain and c-terminal tail of EGFR. Mutation of any of the three cysteine residues to non-palmitoylatable amino acids impeded TKI-induced EGFR dimerization. However, mutation of C797 to serine did not affect TKI-induced EGFR dimerization. The C797S mutation is a major cause of TKI resistance of non-small lung cancers bearing T790M mutation [51]. Given that palmitoylation can also occur at serine residues [39,40], it would be valuable to know whether inhibition of palmitoylation can overcome TKI resistance imposed by C797S mutation.

Resistance to EGFR TKIs are categorically classified into intrinsic and acquired resistance. However, the clinical criteria for intrinsic resistance to TKIs are not adequately defined [52]. All the cancer cell lines used in the study are known to overexpress wt-EGFR with a high basal level of phosphorylation, but their corresponding cancers are intrinsically resistant to TKIs in clinic [9,53,54,55,56]. In this study, we observe that the attenuation of EGFR’s kinase activity had no impact on overall survival of the wt-EGFR-expressing cancer cells. Cell death induced by targeted reduction of EGFR substantiates the fact that that the kinase-inactive EGFR is critical for maintenance of cell survival. In addition, the cytotoxicity associated with disruption of TKI-induced EGFR dimerization by inhibiting palmitoylation indicates that intrinsic resistance is potentially facilitated by EGFR’s dimerization status on the membrane. Recently, it was also demonstrated that TKI-induced EGFR dimerization is a critical mechanism for facilitating acquired resistance in cells harboring EGFR T790M mutation and that targeted reduction of EGFR resulted in tumor regression [57]. All the results combined suggest that TKI-induced kinase-inactivated EGFR dimers have a critical function in sustaining survival of cancer cells in both intrinsic and acquired resistances. In accordance with our previous studies, the pro-survival role of TKI-induced kinase-inactivated EGFR dimers can be attributed to its scaffolding function, which is involved in interacting with and stabilizing other pro-survival proteins [4,27,28,29,30,31,32]. This provides a mechanistic explanation for the lack of therapeutic efficacy associated with TKIs as its main function is to inhibit EGFR’s kinase activity without affecting EGFR’s kinase-independent function. As compared to the parental cells, the TKI-resistant cells exhibited higher sensitivity to palmitoylation inhibition and downregulation of EGFR, suggesting that the chronic TKI treatment might have completely shifted EGFR from its kinase-dependent functions to its kinase-independent pro-survival functions in these cells. Therefore, development of molecular inhibitors that can concurrently target EGFR’s kinase activity, impede dimerization of EGFR, and target EGFR for degradation may be of value to treat EGFR-positive cancers [58].

This study raises some mechanistic questions as to how the kinase-inactivated EGFR facilitates TKI resistance. For example, in response to TKI treatment under both acute and chronic conditions (as depicted in Figure 1C and Figure 2E,F), a portion of EGFR was dimerized but a vast majority of the protein remained as monomers. Further investigation with respect to the functional status and localization of the kinase-inactive dimers, compared to monomers, in TKI-resistant cells is warranted. Previous studies have shown that EGFR on lipid rafts is indicative of resistance and inhibition of lipid raft formations have sensitized EGFR-positive cells to TKI treatments [9,44,59,60,61,62,63,64,65,66]. Therefore, identifying the localization of kinase-inactivated EGFR following TKI treatments and its interaction partners (proteins or protein complexes) will be important in elucidating the pro-survival independent function of kinase-inactivated EGFR and may offer more therapeutic targets for overcoming TKI resistance.

## 4. Materials and Methods

### 4.1. Cell Cultures and Reagents

In this study, we used two separate prostate cancer cell lines (PC3, Du145), lung cancer line (A549), breast cancer line (MDA-MB-231), and non-cancerous cell line (HEK293T). All cells were cultured in dulbecco’s modified eagle medium (DMEM) media containing 5 mM glucose, supplemented with 10% fetal bovine serum (FBS) and 1% antibiotic (penicillin/streptomycin cocktail from Sigma-Aldrich, (St. Louis, MO, USA) Palmitoylation inhibitor 2-bromopalmitate (2-BP, cat# 21604), fatty acid synthase inhibitor (cerulenin, cat# C2389), and EGFR tyrosine kinase inhibitor AEE788 (cat# SML1400) was purchased from Sigma-Aldrich; gefitinib (cat# S1025) and erlotinib (cat# S7786) were purchased from Selleck Bio Antibodies (Houston, TX, USA). Antibodies for EGFR (cat# D38B1) were purchased from Cell Signaling (Danvers, MA, USA). Antibodies for pEGFR (Y1173) were purchased from Invitrogen (Carlsbad, CA, USA) and β-actin (cat# A2228) was purchased from Sigma-Aldrich. Transfections using plasmids and siRNA were conducted using lipofectamine RNAimax (Invitrogen), and polyethylene imine (Sigma-Aldrich).

### 4.2. Establishment of Gefitinib- and Erlotinib-Resistant Cells

Gefitinib resistant (GR) and erlotinib resistant (ER) prostate cancer cells (PC3GR, PC3ER, Du145GR, Du14GR), lung cancer cells (A549GR, A549ER), and breast cancer cells (MD-MBA231GR, MDA-MB-231ER) were developed by chronically treating the corresponding parental cell lines with TKIs. The cells were established by gradually increasing the concentration of TKIs (both gefitinib and erlotinib) to a final concentration of 5 µM for all cells for a period of 3 months in low-glucose (5 mM) medium substituted with 10% FBS and 1% antibiotics.

### 4.3. Plasmids, siRNAs, and Peptide

Plasmids containing wt-EGFR were inserted into PCDNA 3.1 vector as described in our previous publication. The different mutant forms of EGFR including kinase-dead EGFR (R817M), and cysteine mutants of EGFR (C797G) were developed via site-directed mutagenesis as described in our previous papers. The C797S mutant of EGFR were constructed from PCDNA3.1-wt-EGFR-HA by site-directed mutagenesis using Quick Change Lightning site-directed mutagenesis kit from Agilent technologies (cat# 210518). The primers for the C797S mutant were, C797S F: CGCAGCTCATGCCCTTCGGCAGCCTCCTGGACTATGTCCGG and C797S R: CCGGACATAGTCCAGGAGGCTGCCGAAGGGCATGAGCTGCG. The plasmids for other cysteine mutants of EGFR (C1025A and C1122A) on pcDNA 3.1 vector were obtained from Eric S. Witze’s laboratory at the University of Pennsylvania, U.S.A. siRNA for EGFR (cat# EHU076761) was purchased from Sigma-Aldrich. The peptide herdegradin, which was shown to downregulate EGFR in our previous publication, was synthesized at a purity of ˃98% from Genscript Inc. (Piscataway, NJ, USA). Stock concentrations of the peptide were prepared in DMEM medium containing 10% FBS.

### 4.4. Transfections and Western Blotting

To knockdown EGFR in parental (PC3, Du145, A549, and MDA-MB-231) and in corresponding gefitinib- and erlotinib-resistant cells, 1 µg of EGFR siRNA was used per well in 6-well plate format for cytotoxicity assays, and 500 ng of EGFR SiRNA was used in 12-well format for Western blotting purposes. siRNA transfections were carried out using lipofectamine RNAiMax using recommended protocol from Thermo Fisher Scientific (Waltham, MA, USA).

Protein samples from cells for Western blotting were collected in 2 × reduced sample Laemmli buffer. The samples were then denatured in 100 °C for 10 min. An equal amount of protein samples were loaded and resolved on SDS PAGE gel which was then transferred to polyvinylidene fluoride (PVDF) membrane followed by blocking in 5% milk in TBST for 1 h. Different proteins of interest were probed using specific primary and secondary antibodies at optimized concentrations. All samples were subjected to replication three times.

### 4.5. EGFR Dimerization Assay

Plasmids of wild-type and mutant EGFRs (KD, C797S, C797G, C1025A, and C1122A) at a concentration of 100 ng each were transfected into HEK293T cells grown in a 12-well plate format. PEI was used to conduct the transfections. Following 24 h of incubation, the cells were then transferred to serum-free media and treated with DMSO or TKIs (AEE788, gefitinib, and erlotinib) at a concentration of 5 µM each for another 24 h. Cells were then lysed on ice using cold RIPA buffer, containing protease inhibitors, followed by centrifugation to remove cell debris. The cell lysates were then mixed with 2 × non-reduced Laemmli sample (without β-mercaptoethanol) in a 1:1 ratio and EGFR dimers were resolved on a 6% SDS gel followed by Western blotting.

EGFR dimers in reducing conditions were detected using cross-linking agent BS3 (cat# 21580) from Thermo Fischer Scientific. Prostate cancer cell lines (PC3 and Du145), lung cancer cell (A549), and breast cancer cell line (MDA-MB-231) were pretreated with 2-BP (4 µM) or cerulenin (5 µg/mL) in serum-free conditions for 6 h followed by treatment with TKIs (AEE788, gefitinib, and erlotinib) for 24 h. The cells were then washed in ice-cold PBS and incubated with 1 mM BS3 (cat# 21580, Thermo Scientific, (Waltham, MA, USA) for an hour on a shaker at 4 °C. Reaction was quenched using cold PBS and cells lysed using 2 × reduced Laemmli sample buffer (with β-mercaptoethanol). EGFR dimers were then resolved on a 6% SDS PAGE gel followed by Western blotting.

The extent of EGFR dimerization in gefitinib- and erlotinib-resistant cells were also analyzed. The resistant cells were grown to 90% confluency in 12-well formats followed by cross-linking using BS3 using the method described above. The cross-linked samples were resolved on 6% SDS PAGE gel followed by Western blotting.

### 4.6. MTT Cell Proliferation/Cell Viability Assay

Both GR and ER cells (approximately 3 × 10^3^ cells/well) were seeded in 96-well plates. Once adherent, the cells were treated with an increasing concentration of either gefitinib or erlotinib in serum-free media. Cell viability was assessed after 72 h using MTT assay kit from Promega (Madison, WI, USA) (cat# G3582) and procedures followed were according to manufacturer’s protocol. Each treatment condition was triplicated. The data were plotted as percentage of cells that are viable ± SEM.

### 4.7. Trypan Blue Exclusion Assay

EGFR-positive cancer cells (approximately 1 × 10^4^ cells of A549, PC3, Du145, and MDA-MB-231) were seeded on 6-well plates. Once adherent, the cells were treated with an increasing dosage of either gefitinib or erlotinib in serum-free media. The cells were then first washed with PBS followed by trypsinization and subsequent neutralization using DMEM media. The suspended cells were mixed with an equal volume of 0.4% (*w*/*v*) trypan blue (Invitrogen). Both live cells (with clear cytoplasm) and dead cells (stained blue) were counted using a hemocytometer and percent cell viability was calculated ± SD (*n* = 3 for each treatment group).

Both parental and corresponding GR and ER cells were subjected to different treatment conditions in serum-free media for 48 to 72 h in 6-well plate format. The cells were then first washed with PBS followed by trypsinization and subsequent neutralization using DMEM media. The suspended cells were mixed with an equal volume of 0.4% (*w*/*v*) trypan blue (Invitrogen). Both live cells (with clear cytoplasm) and dead cells (stained blue) were counted using a hemocytometer and percent cell death were calculated ± SD (*n* = 3 for each treatment group).

### 4.8. Statistics

All of the data was plotted using GraphPad Prism software. The significance of difference between treatments groups for cytotoxicity assay using trypan blue were determined using one way-ANOVA with Tukey’s multiple comparison post-test within treatment groups. The different levels of significance were labeled with asterisks: * *p* ˂ 0.05, ** *p* ˂ 0.001, **** *p* ˂ 0.0001.

## Supplementary Materials

Supplementary materials can be found at https://www.mdpi.com/1422-0067/20/10/2515/s1.
