# Supplementary material for: Kinase-Inactivated EGFR Is Required for the Survival of Wild-Type EGFR-Expressing Cancer Cells Treated with Tyrosine Kinase Inhibitors
Source: Int J Mol Sci. 2019 May 22;20(10):2515. doi: 10.3390/ijms20102515 (PMC6566606; doi:10.3390/ijms20102515)
Supplement: Supplementary file 1 [file ijms-20-02515-s001.pdf]

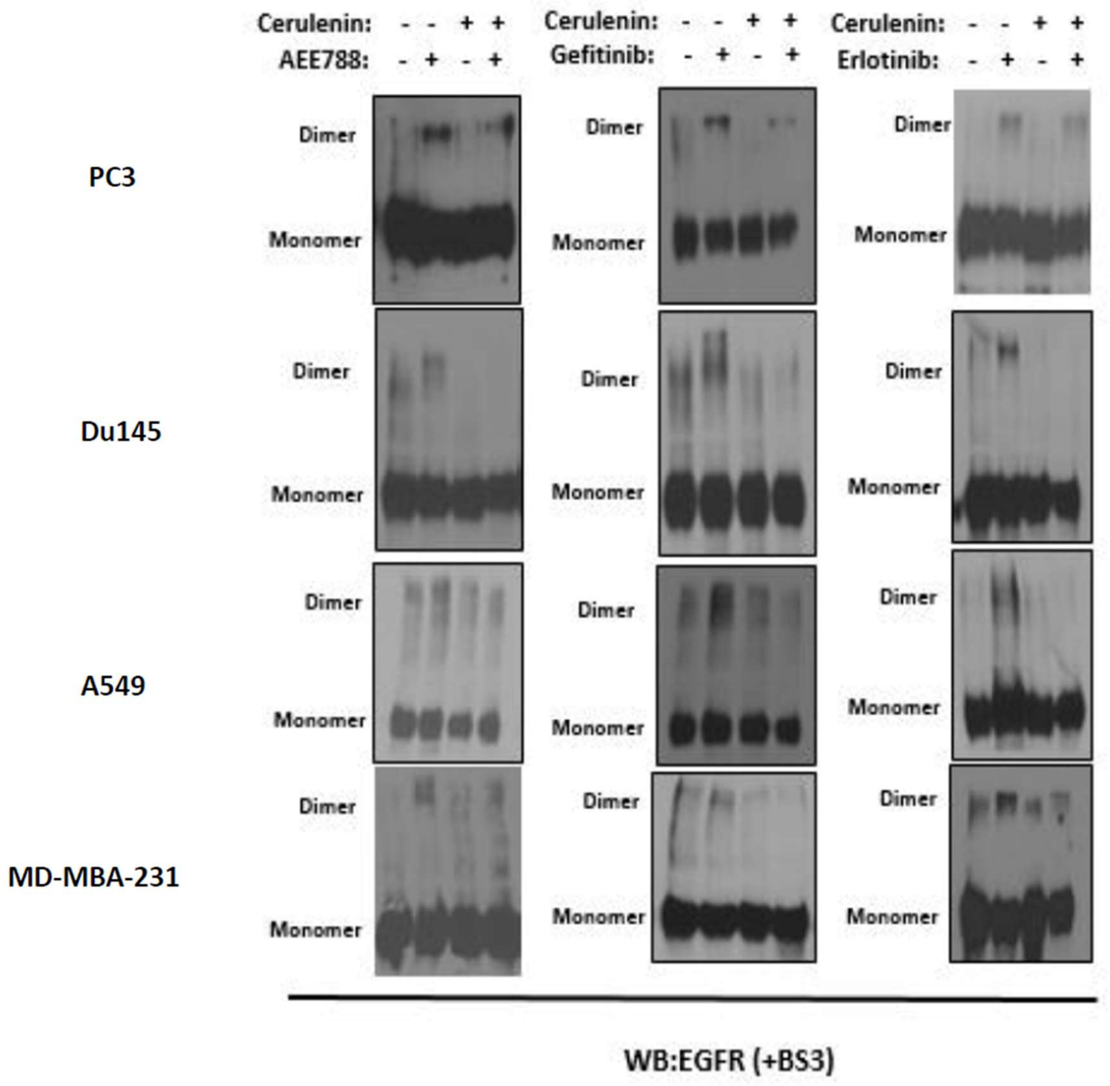

Figure S1. Inhibition of fatty acid synthase-dependent *de novo* palmitate synthesis impedes TKI-induced EGFR dimerization. Cells were pretreated with fatty acid synthase (FASN) inhibitor (cerulenin) at a concentration of 5 μg/mL for 6 h in serum-free media. Following pretreatment, fresh media was added and the cells were treated with respective TKIs (AEE788, gefitinib, and erlotinib) at a final concentration of 5 μM for 24 h. The degree of EGFR dimerization was analyzed following crosslinking using BS3. The cell lysates were resolved on SDS PAGE gel in reducing conditions followed by Western blot.

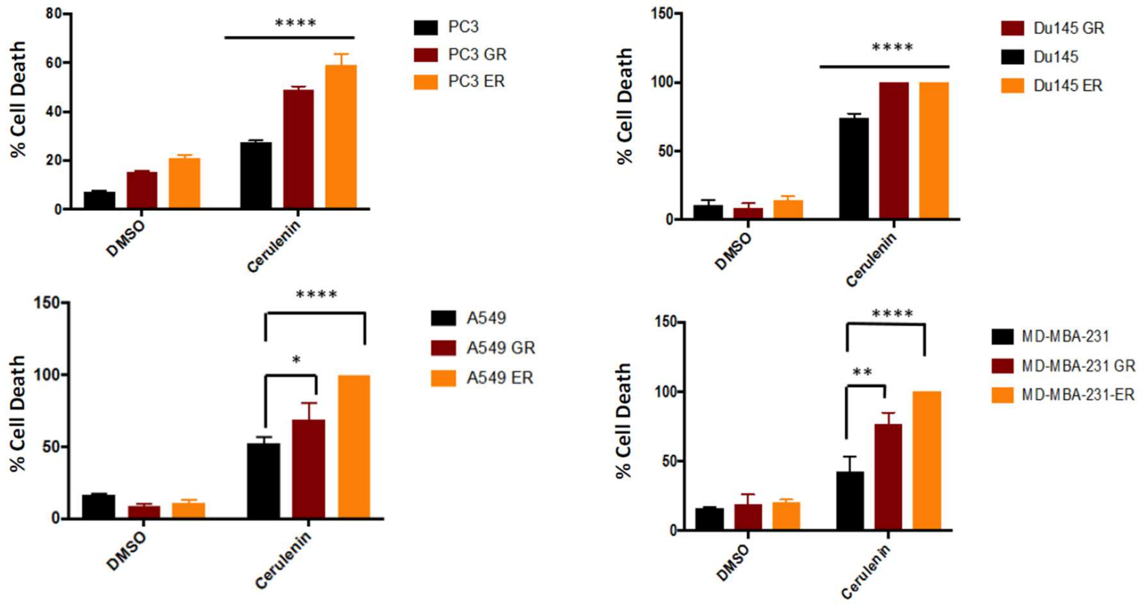

Figure S2. Inhibition of fatty acid synthase increases cytotoxicity in EGFR-TKI-resistant cells. GR and ER cells were seeded on 6-well plates along with respective non-treated parental controls. Once the cells were 80% confluent, the cells were treated with cerulenin (5 μg/mL) for 72 h in serum-free media. Equal volumes of cell suspension was incubated with 0.4% trypan blue to obtain 1:2 dilution. Cells were counted using a hematocytometer and percent cell death calculated. Results were triplicated ± SD and were pooled into treatment groups. One-way ANOVA and Tukey's multiple comparison test was used for statistical analyses, * $p < 0.05$, ** $p < 0.001$, **** $p < 0.0001$.
